# A multiple linear regression analysis identifies factors associated with fear of cancer recurrence in postoperative patients with gastric cancer

**DOI:** 10.1097/MD.0000000000035110

**Published:** 2024-03-15

**Authors:** Xuejuan Zhu, Guijun Ren, Junmin Wang, Yajuan Yan, Xian Du

**Affiliations:** a Gastroenterology Department, The 3rd Hospital of Hebei Medical University, Shijiazhuang, Hebei, P.R. China; b Department of Hepatobiliary Surgery, The 3rd Hospital of Hebei Medical University, Shijiazhuang, Hebei, P.R. China.

**Keywords:** fear of cancer recurrence, gastric cancer, risk factors, social support

## Abstract

To investigate the risk factors of fear of cancer recurrence (FCR) in postoperative patients with gastric cancer (GC) and provide references for targeted nursing intervention development. A total of 84 patients who underwent GC surgery were included in this study. The fear of progression questionnaire-short form and social support rating scale were conducted, and multiple linear regression was performed to identify risk factors of FCR. The score of the fear of progression questionnaire-short form in patients with GC surgery was 39.1 ± 7.6. The results of multiple linear regression showed that age, education level, occupational status, course of the disease, Tumor node metastasis staging, and social support were the influencing factors of FCR in patients with GC (*P* < .05). The current situation of FCR in patients with GC surgery is not optimistic. The medical staff should pay more attention to patients with low age, low education level, unemployment, short course, high tumor node metastasis staging, low social support level, and other high-risk groups, and provide social support resources to reduce the level of FCR.

## 1. Introduction

Gastric cancer (GC) is a common tumor. According to the latest tumor epidemiological investigation in China, the incidence of GC was the second most common, and the mortality rate associated with GC was in third place in China.^[1]^ More than 75% of GC in China represented no apparent symptoms until late in its course, leading to late-stage diagnosis. In recent years, the mortality rate of GC patients has decreased; however, the fear of GC recurrence has become a significant psychological problem with a long-term impact on patients. The recurrence and progression of cancer were the most urgent medical needs which should be solved for cancer patients in China and abroad.^[2]^ Fear of cancer recurrence (FCR) is a psychological state where patients worry about cancer progression or recurrence.^[3]^ Research showed that 39% to 97% of cancer patients had moderate to severe recurrence fear,^[4]^ one of the most common and most painful stress responses in cancer patients.^[5]^ Moderate fear helps patients better resist threats, pay attention to their physical condition, and improve treatment compliance. On the other hand, excessive FCR can develop into chronic posttraumatic stress disorder, affect the prognosis of patients, and seriously affect the patient social and family function.^[6]^ In recent years, there have been many studies on the FCR of breast and cervical cancer at home and abroad. However, few related studies have been reported on the FCR of postoperative GC patients.^[7–9]^ The primary objectives of this study were to explore the risk factors affecting FCR after GC surgery and investigate the incidence of FCR after GC.

## 2. Patients and methods

In this study, all patients received a questionnaire survey from trained nurses. The purpose and significance of the survey were explained to patients before filling out the questionnaire. Acknowledgments of informed consent were obtained from all subjects included in this study. The study was approved by the Ethical Review Board of the Third Hospital of Hebei Medical University.

Patients with GC admitted to the Department of Gastroenterology, 3rd Hospital of Hebei Medical University, from November 2018 to October 2020, were included in the study. Participants were enrolled if they met the following criteria: aged ≥ 18 and met diagnostic criteria for GC by gastroscopy and pathological examination^[10]^; diagnosed with primary GC and treated with surgery or chemotherapy; and had clear consciousness and could express their willingness to participate. Patients were excluded if they had: other malignancies; speech, hearing, or a visual impairment; or previous or current mental illness. Patients who fulfilled the inclusion criteria but refused to participate were also excluded.

## 3. Measures

Variables of interest included sociodemographic variables, including sex, age, marriage, education level, and professional status. Clinical variables included the course of the disease, tumor node metastasis staging (TNM staging), recurrence, metastasis, and social support.

## 4. Survey instrument

### 4.1. The simplified fear scale of disease progression

Single-dimensional simplification by Mehnert,^[11]^ 2006 version, and Wu^[12]^ was conducted for the Sinicization of the scale. The scale consists of 12 items, each scored from 1 to 5, and the total score is 12 to 60. The higher the score, the higher the fear of recurrence. A score ≥ 34 indicates FCR (Cronbach′s α = 0.883).

### 4.2. Social support rating scale

Social support rating scale is a 14-item scale that includes 3 dimensions: objective support, subjective support, and social use of support. The low, medium, and high social support levels were defined as <35, 35 to 45, and > 45 scores, respectively.^[13]^ The Cronbach ɑ scale in this study was 0.865.

### 4.3. Statistical analysis

Independent-sample *t* test, variance analysis, and rank sum test were conducted for univariate analysis on FCR. All tests were performed using SPSS21.0 (IBM, Armonk, NY). Data were presented as mean ± SD for continuous variables. Multiple linear regression was used to analyze the effects of sex, age, degree of education, professional status, TNM staging, course of disease, recurrence or metastasis, and social support on FCR. *P* < .05 was used as the threshold of statistical significance.

## 5. Results

**Figure 1.**
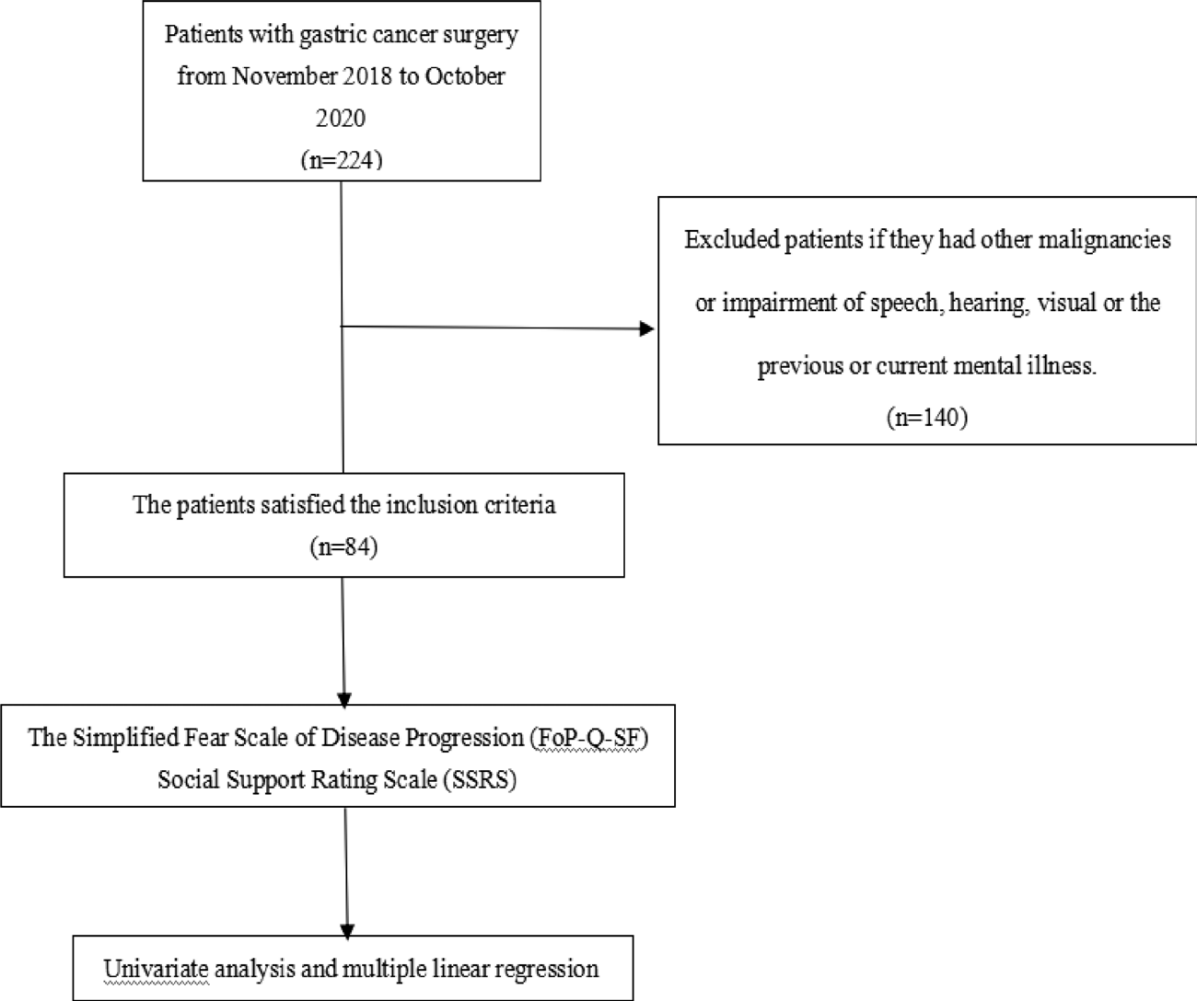
The flow chart for the selection of study participants. FoP-Q-SF = the simplified fear scale of disease progression, SSRS = social support rating scale.

A total of 84 patients with GC that met the inclusion criteria, including 59 males (69.04%) and 25 females (30.95%), were included collected in this study (Fig. 1). The average age was 62.02 ± 7.324 years, ranging from 21 to 84 years. The total score of FCR was 21 to 59, with an average of 39.14 ± 7.64 points. Interestingly, 60 patients (71.42%) had scores ≥34 points.

Univariate analysis showed significant differences in FCR based on sex (*P* < .001), age (*P* < .001), degree of education level (*P* < .001), professional status (*P* < .001), course of disease (*P* < .001), TNM staging (*P* < .001), recurrence or metastasis, (*P* < .001) and social support (*P* < .001) (Table 1). Multiple linear regression analysis was performed, including the above-mentioned analyzed variables as independent variables. The total FCR score was used as the dependent variable. The results showed that age, degree of education, professional status, disease course, TNM staging, and social support significantly affected FCR (Table 2). Patients with low age, low education level, unemployment, short course, high TNM staging, or low social support level had a higher level of FCR.

**Table 1:** Univariate analysis of FCR score in patients after gastric cancer surgery (x̄ ± *s*).

Project	Example number	Score	*t/F*	*P*
Sex				
Male	59	34.19 ± 8.81	−8.826	<.001*
Female	25	42.68 ± 5.37		
Age section				
18~44	16	42.41 ± 6.32	10.205	<.001*
45~60	33	38.83 ± 8.58		
60~75	24	38.75 ± 4.98		
≥75	11	34.96 ± 8.69		
Degree of education				
Primary school and below	15	44.37 ± 3.25	19.499	<.001*
Junior high school and senior high school	23	40.40 ± 6.89		
Junior college	29	36.55 ± 9.37		
Bachelor degree or above	17	35.82 ± 8.22		
Professional status				
Incumbency	63	33.50 ± 5.91	6.724	.001*
other	5	40.26 ± 7.80		
No job	16	41.88 ± 6.32		
Course of disease (yr)				
<1	15	43.56 ± 4.82	28.515	<.001*
1~	12	38.34 ± 10.21		
2~	45	38.24 ± 6.59		
≥3	12	32.87 ± 6.84		
TNM staging				
I	19	35.55 ± 8.54	23.767	<.001*
II	14	35.96 ± 8.84		
III	21	41.46 ± 6.13		
IV	28	44.40 ± 3.52		
Recurrence or metastasis				
Yes	30	43.35 ± 5.57	5.032	<.001*
No	54	38.28 ± 8.06		
Social support				
Low	30	38.65 ± 7.42	65.724	<.001*
Middle	43	43.47 ± 2.43		
High	11	45.50 ± 1.68		

FCR = fear of cancer recurrence, TNM staging = tumor node metastasis staging.

* *P* < .05, it indicates statistically significant differences.

**Table 2:** Multiple linear regression analysis of fear of recurrence in patients after gastric cancer surgery (n = 84).

Factor	B	SE	*β*	*t*	*P*
Sex	−0.484	0.345	−0.071	−1.404	.162
Age	−1.088	0.444	−0.141	−2.452	.015*
Education level	4.700	0.885	0.282	5.312	<.001*
Professional status	−2.966	0.635	−0.227	−4.674	<.001*
TNM staging	−1.800	0.488	−0.275	−3.689	.000*
Course of disease	−0.999	0.355	−0.154	−2.810	.005*
Recurrence or metastasis	−1.310	0.684	−0.083	−1.914	.057
Social support	−9.818	0.644	0.847	15.236	<.001*

TNM staging = tumor node metastasis staging.

* *P* < .05, it indicates statistically significant differences.

## 6. Discussion

### 6.1. The current status of the FCR in patients after GC surgery

Wang et al^[14]^ reported an FCR score of 38.8 ± 5.8 in GC patients, Niu et al^[15]^ reported a score of 36.8 ± 10.7 in breast cancer patients, and Zhen et al^[16]^ reported an FCR score of 25.7 ± 3.2 in patients with lung cancer. This study reported higher FCR levels in postoperative GC patients than in previous reports. This might be because China medical technology level was lagging behind that of European and American countries, and there were still some gaps in the early diagnosis, treatment, and prognosis of cancer compared to developed countries. As an adverse event, when patients were diagnosed with cancer, they were prone to anxiety, depression, and other emotions. Patients developed stress responses and worried that cancer could not be cured despite receiving the treatment.^[17]^ Therefore, the medical staff should take adequate measures to intervene and promote the patients mental health in clinical nursing work.

### 6.2. The risk factors of FCR in patients with GC

Lower age was associated with higher levels of FCR in our study, consistent with previously reported results.^[18]^ This can be explained by the fact that younger patients experience less severe adverse events than seniors, and when faced with sudden stress events, they have little social experience to help them manage their stress. Their good yearning for life was broken, and they lost hope for the future. The young patients had difficulty accepting that they developed cancer, and the fear of separation from parents, spouse, and children, inevitably aggravated the psychological burden and led to a higher psychological fear level. Moreover, the patients were usually the family backbone and provided the household primary income. At the same time, they bore significant social accountability. After illness, the patients would face financial hardship, work disruption, and associated income losses. A study found that the acceptance of commitment therapy could reduce the level of FCR in patients.^[19]^ In clinical care practice, doctors could guide younger patients to translate their potential motivation into practical action to enhance their confidence in the treatment and reduce the level of FCR.

Consistent with a previous study,^[20]^ our results showed a higher FCR level associated with lower education levels. Patients with high education levels had more channels to acquire related knowledge and health literacy, which underpins the remission of inner fear. Patients with inadequate knowledge and understanding of disease would have difficulty resolving their inner fear. Therefore, in clinical nursing practice, patients with low education levels avoid basic medical specialized terms in health education. The language should be colloquial so they can grasp the relevant knowledge of the disease and their conditions, reducing their cancer fear.

This study showed that unemployed patients had higher levels of FCR, which is consistent with the study of Niu et al^[15]^ Patients without occupation had a heavy economic and medical burden because they had unstable income statuses. On the other hand, some patients had mild FCR levels despite job security and steady income. This could be because they think they can return to work after treatment, which might distract patients attention from the disease. Besides, the investment in work gets the patient out of the “disease state” and reduces the psychological level of cancer fear. In clinical nursing, we should pay attention to unemployed patients and use the medical security service system to reduce the FCR.

Longer disease course was linked with higher tumor grade and FCR levels in this study, which is consistent with previous findings.^[21]^ The higher the patient disease stage indicates a higher recurrence rate, leading to lower confidence in treatment. Patients with increased disease courses gradually recover with the appropriate treatment. Furthermore, the recovery effect produces positive psychology in patients, reducing FCR levels. Therefore, in the nursing practice process, patients with advanced disease stages within a 1-year course could be given disease-related knowledge to relieve their confusion and reduce their FCR levels.

This study showed that the higher the social support level, the lower the FCR, which is consistent with the results of Tewari, Mutsaers, and Tomei et al^[22–24]^ High levels of social support play an essential role in the recovery from disease, and it could enable patients to gain psychoemotional support in the face of stressful events, specifically marital relationships.^[25]^ Studies have shown that a couple relationship can help patients learn and adapt to cancer time. Besides, intimacy and a spouse high level of support by revealing their opinion and feelings alleviate the stress level of patients, make them optimistic in the face of disease, and reduce their FCR levels.^[26]^ Therefore, in clinical nursing, patients should be guided to seek social support to reduce FCR.

The significant strengths of this study were the collection of numerous exploratory risk factors for analysis of their association with FCR in postoperative patients with GC. However, there were several potential limitations. First, this was a single-center retrospective study, possibly biasing the accuracy of collected data and the results, although we tried our best to solve that by using data-double-entry and cross-checking. Second, the direct result of postoperative patients with GC was only limited to those occurring during hospitalization, which might have led to underestimating severe adverse complications. Third, the generalizability of our results to other settings still requires further investigation because of its single-center and tertiary referral trauma center design. Fourth, as with every other logistic regression analysis, the confounding effect remains an issue due to the unmeasured, unconsidered, or unquantifiable variables.

## 7. Conclusion

Patients with GC had a high risk of FCR. Age, educational level, occupational status, disease duration, TNM staging, and social support were risk factors of FCR in GC patients. These results could be used in clinical nursing work for risk stratification to reduce recurrence fear in GC patients by providing adequate psychological counseling, targeted measures, and practical social support as soon as possible.

## Acknowledgments

We are grateful to the Medical Records Room of the Third Hospital of Hebei Medical University for providing data.

## Author contributions

**Conceptualization:** Xuejuan Zhu.**Data curation:** Junmin Wang.

**Formal analysis:** Yajuan Yan.

**Investigation:** Xian Du.

**Supervision:** Xuejuan Zhu.

**Validation:** Junmin Wang.

**Visualization:** Yajuan Yan.

**Writing – original draft:** Xuejuan Zhu.

**Writing – review & editing:** Guijun Ren.
